# UPR in palmitate-treated pancreatic beta-cells is not affected by altering oxidation of the fatty acid

**DOI:** 10.1186/1743-7075-8-70

**Published:** 2011-10-06

**Authors:** Ernest Sargsyan, E-ri Maria Sol, Peter Bergsten

**Affiliations:** 1 Department of Medical Cell Biology, Uppsala University, Box 571, SE-75123, Uppsala, Sweden

**Keywords:** beta-cell, human islets, palmitate oxidation, ER stress, unfolded protein response

## Abstract

**Background:**

Elevated levels of lipids are detrimental for beta-cell function and mass. One of the mechanisms of how fatty acids induce apoptosis is development of the unfolded protein response (UPR). It is still far from understood how fatty acids activate the UPR, however.

**Methods:**

We examined how palmitate-induced activation of the UPR was affected by altering the metabolism of the fatty acid in insulin-secreting INS-1E and MIN6 cell lines and intact human islets. To increase oxidation, we used low glucose (5.5 mM) or AICAR; and to reduce oxidation, we used high glucose (25 mM) or etomoxir. UPR was measured after 3, 24 and 48 hours of palmitate treatment.

**Results:**

Modulation of palmitate oxidation by either glucose or the pharmacological agents did not affect palmitate-induced UPR activation.

**Conclusion:**

Our finding suggests that other factors than oxidation of palmitate play a role in the activation of UPR in fatty acid-treated beta-cells.

## Introduction

Extended elevated levels of fatty acids impair glucose-stimulated insulin secretion (GSIS) and induce apoptosis in insulin-secreting beta-cells [1,2]. Various mechanisms of fatty-induced beta-cell apoptosis have been proposed, where development of the unfolded protein response (UPR) has been studied intensely during recent times [3-11]. The UPR or the endoplasmic reticulum (ER) stress response is the adaptive cellular reactions that coordinate down-regulation of overall protein synthesis and increased protein folding capacity by up-regulation of molecular chaperones and enhanced protein degradation [12-15]. Three signaling pathways of UPR, controlled by ER transmembrane proteins PKR-like endoplasmic reticulum kinase (PERK), IRE1 and activating transcription factor (ATF) 6, have been discovered [14]. Under normal conditions, these proteins are inactive due to interaction with molecular chaperone BiP. Accumulation of unfolded proteins leads to dissociation of BiP and activation of these sensors. Activation of PERK occurs early in time and leads to phosphorylation of eukaryotic initiation factor 2α (eIF2α), which attenuates protein synthesis and, at the same time, stimulates translation of ATF4. ATF4 is a transcription factor that regulates expression of molecular chaperones. IRE1, after activation, catalyzes splicing of XBP1. Spliced form of XBP1 encodes an active transcription factor that regulates expression of molecular chaperones and also proteins involved in degradation and secretion. Activation of ATF6 leads to its translocation to Golgi, where after cleavage with proteases it forms an active transcription factor that controls expression of molecular chaperones. When these mechanisms cannot compensate for the ER stress, cell death pathways are activated. The C/EBP-homologous protein/growth arrest and DNA damage-inducible protein (CHOP/GADD153) transcription factor and JNK have been implicated in this aspect of the UPR [16]. Proposed mechanisms of how fatty acids induce ER stress include ER Ca^2+ ^release, overload of ER with unfolded proteins and accumulation of tripalmitin in the ER [17,18]. In the present study, we examined the role of palmitate metabolism in the fatty acid-triggered activation of UPR in insulin-secreting cell lines INS-1E and MIN6 and intact human islets. It is known that reduced oxidation of palmitate directs long chain CoA towards non-oxidative metabolic pathways, such as generation of TAGs and ceramides [19,20]. Palmitate oxidation was modified by using various concentrations of glucose. According to the malonyl-CoA hypothesis high glucose reduces fatty acid oxidation, which is due to inhibition of fatty acid transporter CPT1 by increased production of malonyl-CoA [19,20]. At low glucose, beta-cells oxidize fatty acids [21]. Additionally, we used AMPK agonist AICAR, which favors fatty acid oxidation and prevents lipotoxicity, and CPT1 inhibitor etomoxir that reduces fatty acid oxidation and aggravates lipotoxicity [19,22,23].

## Materials and methods

### Cell culture

Rat INS-1E cells (a kind gift from Dr. Pierre Maechler, Geneva University) were cultivated in RPMI 1640 medium containing 11 mM glucose and supplemented with 10% fetal bovine serum (FBS), 2 mM L-glutamine, 1 mM sodium pyruvate, 10 mM HEPES and 55 μM β-mercaptoethanol at 37°C and 5% CO_2_. All reagents were purchased from Invitrogen (Carlsbad, CA). Experiments with INS-1E cells were performed between passages 65 and 90. Mouse insulinoma MIN6 cells (a kind gift from Prof. Jun-Ichi Miyazaki, Osaka University) were maintained in Dulbecco's Modified Eagle medium (DMEM) containing 25 mM glucose and supplemented with 10% FBS and 55 μM β-mercaptoethanol at 37°C and 5% CO_2_. All experiments with MIN6 cells were performed between passages 21 and 28. Human islets were obtained from the Islet Transplantation Unit at Uppsala University from non-diabetic individuals. Human islets were cultured in CMRL 1066 medium containing 5.5 mM glucose and supplemented with 10% FBS.

### Ethics Statement

Ethical permission to use human islets isolated from healthy individuals have been obtained from the Regional Ethical Review Board in Uppsala (date: 2010-02-10; number 2010/006).

### Free fatty acid preparation and cell/islet treatment

Culture medium containing palmitate (Sigma, St. Louis, MO) was prepared as previously described [4,10]. Briefly, the fatty acid was dissolved in 50% ethanol to a concentration of 100 mM. This stock solution was diluted in culture medium to a required concentration and then allowed to complex with 0.5% fatty acid free BSA (Boehringer Mannheim GmbH, Mannheim, Germany) for 30 min at 37°C. Cells cultured to 65-70% confluence or ~50 human islets were exposed to palmitate in the presence of different concentrations of glucose for 48 hours. Whereas FBS was maintained during palmitate exposure of INS-1E and MIN6 cells [10,11], FBS was removed during palmitate incubation of human islets [24]. Cells and islets cultured in the presence of palmitate were also treated with 1 mM AICAR or 0.2 mM etomoxir (both purchased from Sigma).

### Measurements of palmitate oxidation rate

Cells/islets were cultured in media containing 0.5 mM palmitate and 2 μCi [^3^H]palmitate per ml. To measure oxidation, media samples were collected after culture and ^3^H_2_O separated from [^3^H]palmitate using Folch extraction [25]. The volume of 10 ml Ultima Gold™ scintillation fluid (Chemical Instruments AB, Sollentuna, Sweden) was added to 500 μl ^3^H_2_O and radioactivity determined by a liquid-scintillation spectrometer (Wallac System 1400™ PerkinElmer, Boston, MA). The results were normalized to DNA content.

### Protein measurements by Western blot analysis

Details of the procedure for immunoblotting have been described previously [26]. Immunoblot analyses were performed with antibodies towards phosphorylated PERK (p-PERK), phosphorylated eIF2α (p-eIF2α) (Cell Signaling, Beverly, MA), CHOP/GADD153 (Santa Cruz Biotechnology, Santa Cruz, CA) and BiP/GRP78 (Abcam, Cambridge, UK) [27]. Immuno-reactive bands were visualized with Fluor-S MultiImager MAX (BioRad, Hercules, CA) and quantified with Quantity One software (Bio-Rad).

### Analysis of mRNA expression by real-time PCR

Total mRNA was isolated from the cells by Trizol (Invitrogen) and reversely transcribed with SuperScript™ III First-Strand Synthesis System for RT-PCR (Invitrogen). The real-time PCR was performed in 10 μl volume containing ~20 ng cDNA, 0.5 μM forward and reverse primers and 5 μl Dynamo Capillary SYBR Green qPCR kit (Finnzymes, Espoo, Finland). Primers used for the amplification are shown in Table 1. PCR products were quantified fluorometrically using SYBR Green and normalized to the housekeeping gene β-actin and relative to the control (11 mM glucose). The following formula was used: target amount = 2^-ΔΔ*Ct*^, where ΔΔ*Ct *= {[*Ct *(target gene sample) - *Ct *(*β-*actin sample)] - [*Ct *(control sample) - *Ct *(*β-*actin control)} [28].

**Table 1:** Primers used for real-time PCR

Target	Forward primer	Reverse primer
b-actin	5'-TCTGTGTGGATTGGTGGCTC-3'	5'-GACTCATCGTACTCCTGCTTGCT-3'

CHOP/GADD153	5'-CCAGCAGAGGTCACAAGCAC-3'	5'-CGCACTGACCACTCTGTTTC-3'

GADD34	5'-GTCCATTTCCTTGCTGTCTG-3'	5'-AAGGCGTGCCCATGCTCTGG-3'

ATF4	5'-GTTGGTCAGTGCCTCAGACA-3'	5'-CATTCGAAACAGAGCATCGA-3'

Spliced XBP1 rat	5'-GAGTCCGCAGCAGGTG-3'	5'-GCGTCAGAATCCATGGGA -3'

EDEM	5' -CAGTCAAGTTAGTGATCAACAC- 3'	5' - TGTCATGTCACCAAAGGGCTG - 3'

### Statistical analysis

Results are presented as means ± SEM. Statistical significance between two conditions was analyzed using one-way ANOVA with Tukey post-hoc test. *P *< 0.05 was considered statistically significant.

## Results

### Palmitate oxidation is reduced by high glucose in human islets, MIN6 cells and INS-1E cells

**Figure 1:**
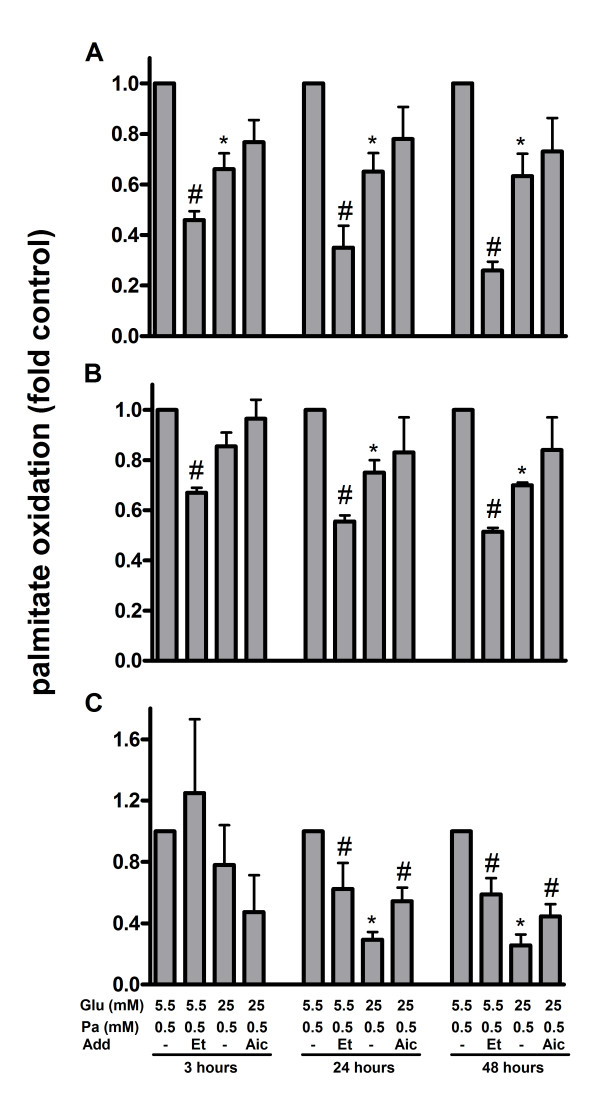
**Palmitate oxidation in palmitate-exposed human islets (A), MIN6 cells (B) and INS-1E cells (C)**. The cells and islets were cultured for 3, 24 and 48 hours in the presence of 0.5 mM palmitate (Pa) and 2 μCi [^3^H]palmitate per ml at 5.5 or 25 mM glucose (Glu). AICAR (1 mM; Aic) or etomoxir (0.2 mM; Et) were added as indicated. Islets cultured at 5.5 mM glucose; MIN6 cells cultured at 25 mM glucose and INS-1E cells cultured at 11 mM glucose alone were considered as controls. After culture, media were collected, ^3^H_2_O separated and radioactivity determined by a liquid-scintillation spectrometer. Results are means ± SEM of 3-4 independent experiments. *P < 0.05 compared to low glucose and #p < 0.05 effect of additive.

Palmitate oxidation was measured in palmitate-exposed human islets, MIN6 and INS-1E cells cultured at 5.5 (low) or 25 (high) mM glucose for 3, 24 or 48 hours. In human islets (Figure 1A) and MIN6 cells (Figure 1B) cultured at high glucose palmitate oxidation was lowered by about 30% compared to islets and cells cultured at low glucose. In human islets reduced oxidation of the fatty acid in the presence of high glucose compared to low glucose was already detected after 3 hours. In INS-1E cells exposed to palmitate for 24 or 48 hours oxidation rate of the fatty acid was reduced by 80% in cells cultured at high glucose compared to cells cultured at low glucose (Figure 1C). After 3 hours, palmitate oxidation in INS-1E cells cultured at low and high glucose was similar. Addition of AICAR partially restored the glucose-dependent reduction in palmitate oxidation in INS-1E cells (Figure 1C) but not in human islets and MIN6 cells (Figures 1A and 1B). When etomoxir was added palmitate oxidation was reduced in both cell lines and islets (Figure 1).

### Palmitate-induced UPR is not affected by high glucose in human islets, MIN6 cells or INS-1E cells

**Figure 2:**
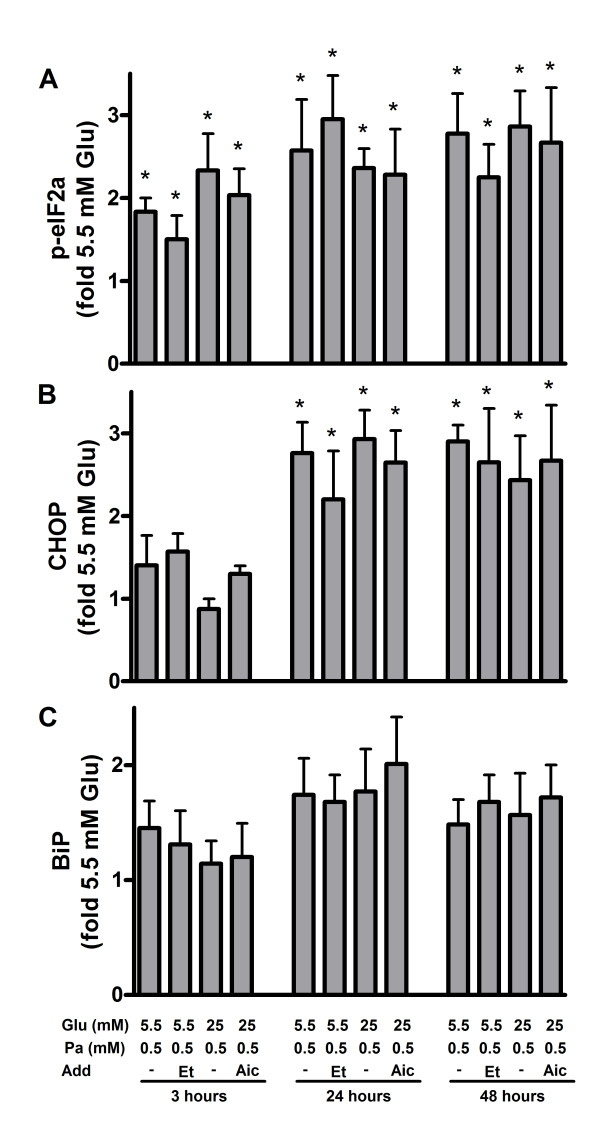
**Unfolded protein response in palmitate-exposed human islets**. The islets were cultured for 3, 24 or 48 hours in the presence of 0.5 mM palmitate (Pa) at 5.5 or 25 mM glucose (Glu). AICAR (1 mM, Aic) or etomoxir (0.2 mM, Et) were added as indicated. Islets cultured at 5.5 mM glucose alone were considered as control. After culture, protein levels of p-eIF2α (A), CHOP (B) and BiP (C) were measured. Protein levels are means ± SEM of 4 independent experiments.*P < 0.05 compared to control.

**Figure 3:**
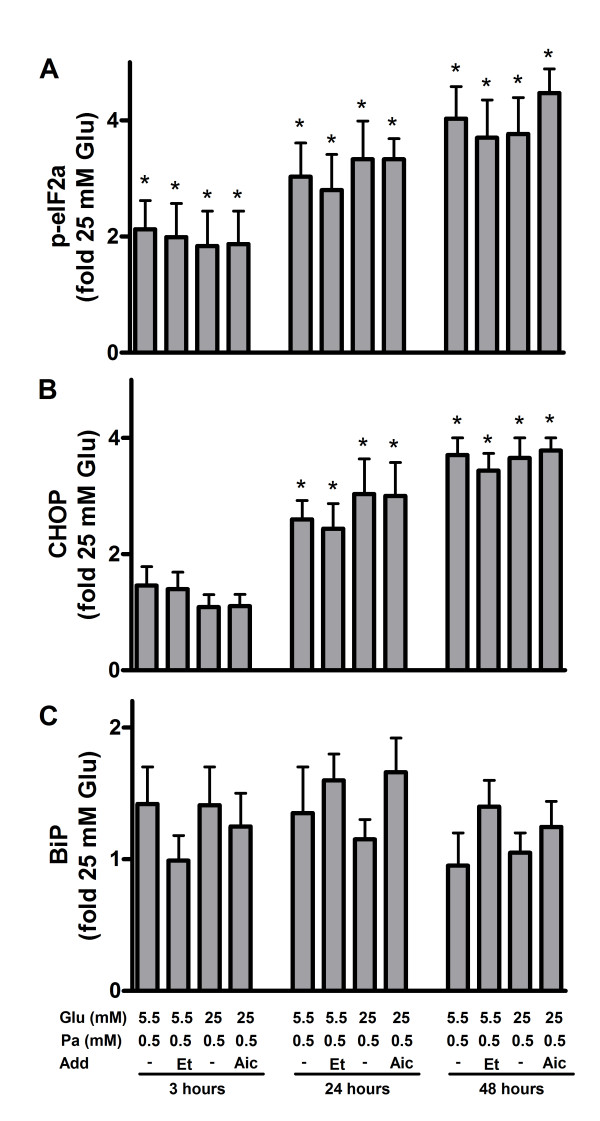
**Unfolded protein response in palmitate-exposed MIN6 cells**. The cells were cultured for 3, 24 and 48 hours in the presence of 0.5 mM palmitate (Pa) at 5.5 or 25 mM glucose (Glu). AICAR (1 mM, Aic) or etomoxir (0.2 mM, Et) were added as indicated. Cells cultured at 25 mM glucose alone were considered as control. After culture, protein levels of p-eIF2α (A), CHOP (B) and BiP (C) were measured. Protein levels are means ± SEM of 4 independent experiments. *P < 0.05 compared to control.

Translational attenuation is an early event of the UPR and is achieved via the phosphorylation of PERK and eIF2α [29]. In human islets and MIN6 cells exposed to palmitate levels of p-eIF2α rose approximately 2-fold after 3 hours and continued to rise in MIN6 cells (P < 0.05) but not in islets up to 48 hours (Figures 2A and 3A; additional files 1A and 1B). Levels of p-eIF2α levels in human islets and MIN6 cells exposed to palmitate were neither changed by altering the glucose concentration nor by adding AICAR or etomoxir. If ER stress is not alleviated, activation of the PERK pathway leads to up-regulation of pro-apoptotic protein CHOP [30]. When protein levels of CHOP were measured in human islets and MIN6 cells, no alterations were observed after 3 hours (Figures 2B and 3B; additional files 1A and 1B). After 24 and 48 hours, palmitate caused 2-3 fold induction in the expression level of CHOP, however. The induction was neither dependent on glucose nor etomoxir or AICAR concentrations. Potential activation of IRE1 and ATF6 signaling was examined by measuring expression level of BiP in human islets and MIN6 cells exposed to palmitate. Levels of the chaperone were not altered irrespective of culture time, glucose, AICAR or etomoxir concentrations (Figures 2C and 3C; additional files 1A and 1B).

**Figure 4:**
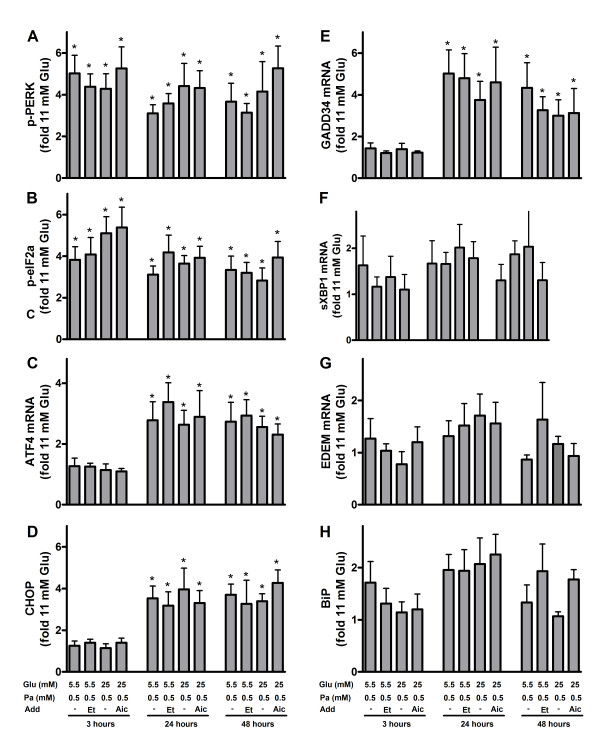
**Unfolded protein response in palmitate-exposed INS-1E cells**. The cells were cultured for 3, 24 or 48 hours in the presence of 0.5 mM palmitate (Pa) at 5.5 or 25 mM glucose (Glu). AICAR (1 mM, Aic) or etomoxir (0.2 mM, Et) were added as indicated. Cells cultured at 11 mM glucose alone were considered as control. After culture, protein levels of p-PERK (A), p-eIF2α (B), CHOP (D), BiP (H) and transcript levels of ATF4 (C), GADD34 (E), sXBP1 (F) and EDEM (G) were measured. Protein and transcripts levels are means ± SEM of 6 independent experiments. *P < 0.05 compared to control.

Difference in palmitate oxidation observed at low and high glucose was especially marked in INS-1E cells (Figure 1A). UPR in INS-1E cells was therefore studied more thoroughly. When phosphorylation levels of eIF2α were measured in INS-1E cells cultured in the presence of palmitate, p-eIF2α levels rose approximately 5-fold already after 3 hours and remained elevated after 24 and 48 hours (Figure 4B; additional file 1C). The degree of palmitate-induced phosphorylation of eIF2α was neither affected by the glucose concentration, nor inclusion of AICAR or etomoxir, however. The lack of glucose-dependency made us also measure phosphorylation levels of PERK, the kinase responsible for phosphorylation of eIF2α [14]. Phosphorylation patterns of PERK were almost identical to those observed of p-eIF2α (Figure 4A; additional file 1C). Translation of transcription factor ATF4 is controlled by p-eIF2α. When transcript level of ATF4 was measured, no significant changes were observed after 3 hours. After 24 and 48 hours, palmitate caused 3-fold induction in the expression level of ATF4 (Figure 4C). Again, these alterations were independent on glucose concentration or addition of AICAR or etomoxir. CHOP is a downstream target of ATF4. It has pro-apoptotic properties and regulates expression of another apoptotic factor, GADD34 [31]. Expression level of CHOP transcript (not shown) and protein (Figure 4D; additional file 1C) as well as GADD34 transcript (Figure 4E) were very similar to that of ATF4. When IRE1 and ATF6 signaling were examined in INS-1E cells exposed to palmitate, no significant alterations in the expression level of pathway markers, sXBP1 (Figure 4F), EDEM (Figure 4G) and BiP (Figure 4H; additional file 1C), were observed irrespective of time, glucose, AICAR or etomoxir concentrations.

When markers of the UPR were compared with palmitate oxidation in INS-1E cells, MIN6 cells or human islets, no correlation was observed.

## Discussion

ER stress and activation of UPR has been suggested as a mechanism of how raised nutrient levels exert their detrimental effects on palmitate-treated beta-cells. Support for a role of ER stress in lipotoxicity via activation of the UPR has been supplied by studies, where manifestations of the UPR were recorded in beta-cells undergoing apoptosis as a consequence of exposure to palmitate [4-7,9,10]. In triggering ER stress saturated fatty acids such as palmiate are much more potent than unsaturated fatty acids, such as oleate [32]. In line with this, knocking-down SCD1 in palmitate-treated beta-cells aggravates the toxic effect of the saturated fatty acid [33].

The mechanisms of how fatty acids induce ER stress are yet unclear. Some authors hypothesize that prolonged exposure to palmitate causes ER Ca^2+ ^release, which disturbs ER homeostasis and causes ER stress [24,34]. However, it is unlikely that the observed modest depletion of ER Ca^2+ ^may lead to ER stress [4]. Another argument against this hypothesis is that release of ER Ca^2+ ^was observed in cells treated with oleate, which does not induce ER stress [24,34]. ER protein overload is another mechanism, which may contribute to ER stress in lipotoxic beta-cells. Recent studies showed that protein overload might be a consequence of reduced ER-to-Golgi protein trafficking or degradation of carboxypeptidase E [35,36]. However, absence of significant changes in the expression level of molecular chaperones, such as BiP, in the current and previous studies makes this hypothesis questionable [4,10,11]. Up-regulation of ER chaperones is a major response to accumulation of unfolded protein in the ER. The protective role of BiP is also questioned in the study, where over-expression of BiP in INS-1 cells and MIN6 cells partially reduced susceptibility to thapsigargin but failed to reduce palmitate-induced ER stress [6]. Furthermore, gene expression analysis did not detect changes in mRNA level of ER chaperones in palmitate-treated beta-cells [37]. It should also be mentioned that in some studies results concerning expression and role of BiP in palmitate-treated beta-cells are opposite. Thus, Kharroubi et al. demonstrated palmitate-induced up-regulation of BiP mRNA level connected with enhanced apoptosis [5]. Also, Laybutt et al. observed protective, ER stress-reducing and anti-apoptotic effects of BiP over-expression [7].

Prolonged exposure to palmitate leads to formation of tripalmitin [18]. It was shown that tripalmitin accumulates in the ER rather than in the cytoplasm of the beta-cells. This is in contrast to oleate, which forms droplets in the cytoplasm [18]. Accumulation of insoluble tripalmitin may cause morphological perturbations in the ER and be toxic for the beta-cells [17,18]. We hypothesized that reducing fatty acid oxidation would accentuate ER stress by increasing generation and incorporation of tripalmitin into microsomes. To reduce metabolism we raised glucose, which inhibits palmitate oxidation and shuttles the fatty acid to non-oxidative pathways [20,23]. Also, we used AMPK activator AICAR that stimulates, and CPT1 inhibitor etomoxir that inhibits oxidation [19,22,23]. Fatty acid oxidation was higher at low glucose compared to high glucose concentration both in cell lines and human islets, but especially pronounced in INS-1E cells. Accentuated glucose-induced reduction in palmitate oxidation in INS cells was also observed in previous studies [23,38]. More efficient oxidation of fatty acids by MIN6 cells and human islets compared to INS-1E cells in the presence of high glucose might be explained by higher activity of ACC in INS-1E cells [23]. The accentuated glucose-induced reduction in palmitate oxidation in INS-1E cells may explain the significant effect of AICAR in these cells. Surprisingly, essentially similar activation of UPR by palmitate was observed both at low and high glucose concentrations. Furthermore, stimulation and inhibition of palmitate oxidation by AICAR and etomoxir did not affect palmitate-induced UPR activation. This holds true for both cell lines and human islets. More robust activation of UPR markers in INS-1E cells may speak in favor of higher sensitivity of these cells to palmitate exposure.

Previous data on the role of glucose and fatty acid metabolism in palmitate-induced ER stress response are controversial. While Bachar et al claimed that glucose amplified ER stress [39], Cunha et al showed that glucose did not amplify ER stress in palmitate-treated beta-cells [24]. The discrepancy was suggested to be explained by the fact that protein and mRNA levels of ER stress markers were analyzed by the respective groups. Inhibition of CPT1 by siRNA approach performed by Choi et al caused increased expression of CHOP but decreased phosphorylation of eIF2α and JNK [40].

## Conclusions

Our finding suggests that factors other than palmitate oxidation play major role in the palmitate-induced activation of UPR. Furthermore, the fact that apoptosis in beta-cells depends on the oxidation of palmitate suggests that the UPR is not a major mechanism by which the fatty acid negatively affects the beta-cell. The mechanisms by which palmitate activates UPR and its role in the induction of apoptosis requires additional studies.

## Competing interests

The authors declare that they have no competing interests.

## Authors' contributions

ES participated in the design of the study, carried out all the studies, analyzed the data and drafted the manuscript. EMS performed some WB experiments. PB helped to draft the manuscript. All authors have read and approved the final manuscript.

## Supplementary Material

Additional file 1 **Representatives gels of WB in human islets (A), MIN6 cells (B) and INS-1E cells (C)**. The cells and islets were cultured for 3 and 48 hours in the presence of 0.5 mM palmitate (Pa) at 5.5 or 25 mM glucose (Glu). AICAR (1 mM; Aic) or etomoxir (0.2 mM; Et) were added as indicated. Islets cultured at 5.5 mM glucose, MIN6 cells cultured at 25 mM glucose and INS-1E cells cultured at 11 mM glucose alone were considered as controls. After culture, proteins were isolated and subjected to immunoblot analyses with antibodies towards p-PERK (C), p-eIF2α (A; B; C), CHOP (A; B; C) and BiP (A; B; C). Click here for file
