# Supplementary figures and images for: UPR in palmitate-treated pancreatic beta-cells is not affected by altering oxidation of the fatty acid
Source: Nutr Metab (Lond). 2011 Oct 6;8:70. doi: 10.1186/1743-7075-8-70 (PMC3197479; doi:10.1186/1743-7075-8-70)

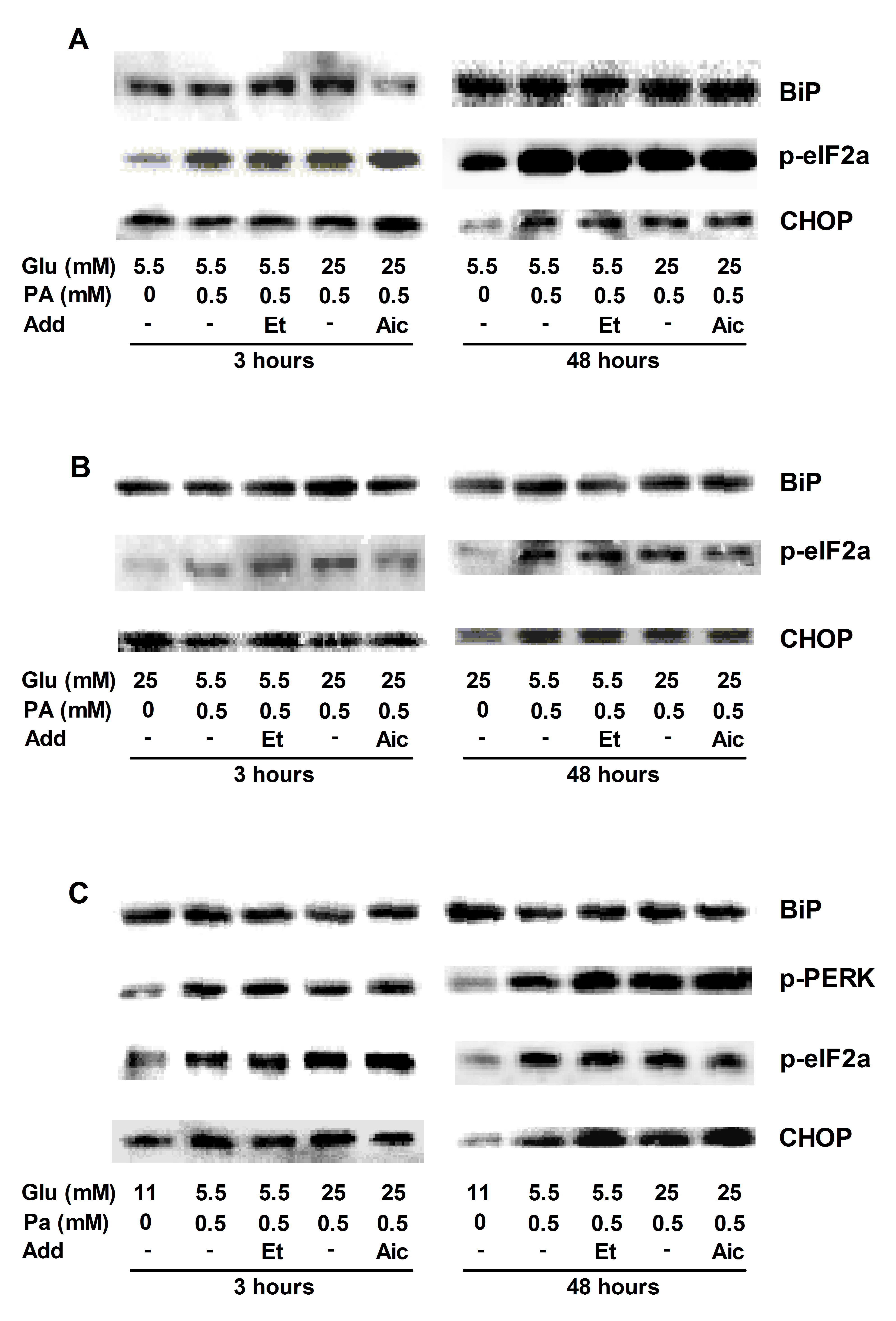

Supplement: Additional file 1 — Representatives gels of WB in human islets (A), MIN6 cells (B) and INS-1E cells (C). The cells and islets were cultured for 3 and 48 hours in the presence of 0.5 mM palmitate (Pa) at 5.5 or 25 mM glucose (Glu). AICAR (1 mM; Aic) or etomoxir (0.2 mM; Et) were added as indicated. Islets cultured at 5.5 mM glucose, MIN6 cells cultured at 25 mM glucose and INS-1E cells cultured at 11 mM glucose alone were considered as controls. After culture, proteins were isolated and subjected to immunoblot analyses with antibodies towards p-PERK (C), p-eIF2α (A; B; C), CHOP (A; B; C) and BiP (A; B; C). [file 1743-7075-8-70-S1.JPEG]
